# Can Current Regulations Account for Intentionally Produced Nanoplastics?

**DOI:** 10.1021/acs.est.2c00965

**Published:** 2022-03-14

**Authors:** Fazel Abdolahpur Monikh, Steffen Foss Hansen, Martina G. Vijver, Esther Kentin, Maria Bille Nielsen, Anders Baun, Kristian Syberg, Iseult Lynch, Eugenia Valsami-Jones, Willie J.G.M. Peijnenburg

**Affiliations:** † Department of Environmental & Biological Sciences, University of Eastern Finland, FI-80101 Joensuu, Finland; ‡ Department of Experimental Limnology, Leibniz Institute for Freshwater Ecology and Inland Fisheries, Müggelseedamm 310, 12587 Berlin, Germany; § Department of Environmental Engineering, Technical University of Denmark, Bygningstorvet Bygning 115, 2800 Kgs. Lyngby, Denmark; ∥ Institute of Environmental Sciences (CML), Leiden University, Einsteinweg 2, 2333 CC Leiden, The Netherlands; ⊥ Institute for the Interdisciplinary Study of the Law, Leiden University, Steenschuur 25, 2311 ES Leiden, The Netherlands; # Department of Science and Environment, Roskilde University, Universitetsvej 1, 4000 Roskilde, Denmark; ¶ School of Geography, Earth and Environmental Sciences, University of Birmingham, Edgbaston, B15 2TT Birmingham, United Kingdom; ∇ National Institute of Public Health and the Environment (RIVM), Center for Safety of Substances and Products, Bilthoven 3720 BA, The Netherlands

**Keywords:** intentionally produced nanoplastics, regulatory frameworks, plastic pollution, risk assessment, risk governance

Regulation of plastics has emerged as a significant science-policy challenge, initiated by the increasing societal concerns regarding plastic pollution. A specific focus is now on plastic of sizes smaller than 1000 nm, often referred to as nanoplastics. The need to include nanoplastics in existing regulatory frameworks arises from the increased bioavailability and toxicity of smaller particles compared to larger fragments.^1^ The size of particles plays a critical role in their uptake and influences particle reactivity and hazard potential, for example, production of reactive oxygen species. When considering nanoplastics, specific concerns are directed to size fractions of plastics <100 nm, consistent with the size-specific concerns regarding ultrafine particles and with the regulatory definition of nanomaterials. Thus, classification of nanoplastics as “nanomaterials” for regulatory purposes would seem logical. However, as nanoplastics consist mainly of polymers, they could also be regulated as polymers, which are currently exempted from registration under REACH (Registration, Evaluation, Authorisation and Restriction of Chemicals). It is also noteworthy that the vast majority of micro- and nanoplastics in the environment arise from the weathering of plastic waste and as such would not be addressed under existing chemicals legislation, even if plastics required registration.

Currently, three important policy and legislative processes are ongoing in parallel that will impact the future regulation of plastics in general and intentionally produced micro- and nanoplastics in particular. First, the European Commission (EC) is considering the restriction proposal commissioned to the European Chemicals Agency (ECHA) on intentionally added microplastics.^2^ Second, there is a discussion about how to define polymers under the European Chemicals Regulation, REACH, so that polymers are not automatically exempted from registration and submission of health and environmental safety information. Third, the EC is currently revising their proposed definition of nanomaterials. Here, we focus on regulatory concerns related to intentionally produced nanoplastics and outline how the inclusion of these three aspects could impact the future regulation of nanoplastics.

## Regulating Nanoplastics As Microplastics

In January 2019, ECHA submitted a proposal on restriction of intentionally added microplastics to avoid or reduce environmental release of microplastic.^3^ In the first version of the proposal, “microplastic” entailed polymers in the size range from 1 nm to 5 mm. However, this size range was questioned during the public consultation. Several stakeholders argued that the lower size limit of 1 nm would not be possible to enforce due to the lack of proper analytical methods to detect and quantify polymers smaller than 100 nm.^4^ ECHA, thus, revised the proposal changing the lower size limit to 100 nm, arguing that this is “a pragmatic solution that balances risk reduction against the obvious analytical constraints and challenges of the initially proposed 1 nm limit”. ECHA further noted that microplastics smaller than 100 nm that are possible to reliably characterize should not be intentionally added to products.^5^ ECHA’s Risk Assessment Committee recommended defining microplastics without a specific lower size limit as there is no clear scientific basis, in terms of hazard, for determining such a limit.^5^ The Socio-economic Analysis Committee opinion recommends that the microplastic definition should contain a lower size limit of 1 nm but recognizes that a temporary lower size limit of 100 nm in the restriction conditions is required in order for the restriction to be “implementable, enforceable and monitorable “.^5^

Analytical limitation might not be a reasonable justification for leaving out intentionally added nanoplastics from microplastic regulations. The main challenge associated with analyzing nanoplastics is related to their diversity of size, chemical composition, and purity when they occur in the environment alongside other organic materials with similar chemistry and size distributions.^6^ Determining the presence of nanoplastics in products might not create such an analytical challenge for their quantification, considering that specific sizes are used, and that the composition of the particles and the products should already be known. Moreover, remarkable progress has been made in terms of nanoplastic identification and quantification.^6^ For example, thermal desorption–gas chromatography mass spectrometry^7^ and dynamic light scattering following extraction of the particles from product matrices^8^ can be used for the identification and quantification of nanoplastics in products. Thus, intentionally added plastic particles in the nanorange (1–100 nm) could be reintroduced into the restriction proposal.

## Regulating Nanoplastics As Nanomaterials

In principle, the size range for engineered nanomaterials (1–100 nm) was initially defined to enable safety assessment and management of intentionally produced nanoscale objects whose properties, and potential toxicity, are distinct from their larger counterparts. It is noteworthy that polystyrene nanoplastics were included in the original list of high-volume industrially relevant nanomaterials for assessment by the OECD (Organisation for Economic Co-operation and Development) Sponsorship program for the testing of manufactured nanomaterials but was removed in the 2010 revision of the materials and end-points on the basis of updated knowledge on production and use and lack of commercial relevance.^9^

In 2011, the EC adopted a recommended definition for nanomaterials, and the revised definition will be published shortly.^10^ According to the EC definition plastic particles would be defined as nanomaterials if 50% or more of the plastic particles in the number size distribution are within the size range 1–100 nm. If such intentionally added nanoplastics are indeed considered as nanomaterials in a regulatory context, nanospecific data requirements will apply. Such requirements are already in place in some EU regulations and directives, for instance, the Cosmetics Regulation, the Biocidal Product Regulation (BPR), and Food Contact Materials (FCMs) legislation. The relevance of the legal provisions of the Cosmetics Regulation, the BPR and the FCMs depends on whether the used polymers fall under the scope of the specific regulation, for example, biocidal active substance, or cosmetic ingredient. In 2020, an Annex was added to REACH that requires manufacturers and importers to register “nanoforms” and to demonstrate the safety of all their potential uses. It has been pointed out that many of the test-specific requirements, as well as options for data waiving, depend on subjective terms such as “poorly soluble”, “high insolubility”, and “low” versus “high” dissolution rates.^11^ If included in REACH, it is likely that most nanoplastics would be considered poorly soluble.

## Regulating Nanoplastics As Polymers

Polymers are currently exempted from registration under REACH as they are considered to be of low concern, due to their high molecular weight and (assumed) correlated lower (eco)toxicological concerns. In accordance with REACH, a “polymer molecule” is a molecule that contains a sequence of at least three monomer units, which are covalently bound to at least one other monomer unit or other reactant. Currently the EC is developing a proposal to initiate the polymer registration process under REACH. The EC’s proposed criteria for identification of polymers requiring registration under REACH^12^ has been criticized by experts as covering only a small percentage of the polymer types estimated to be available on the EU market,^13^ and for excluding the main polymer responsible for plastic pollution, that is, polyethylene. The final decision on how polymers shall be registered is yet to be released by ECHA or the EC.

**Figure 1:**
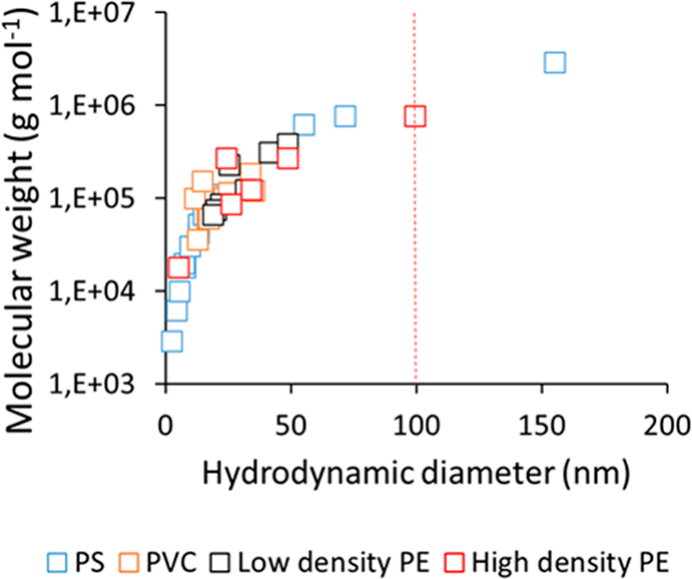
Mw of polymers (PS: polystyrene, PVC: polyvinyl chloride, PE: polyethylene) versus their calculated/reported *R*_h_ (in Tetrahydrofuran at 25 °C). The dashed red line shows the defined upper limit of nanomaterials proposed for regulatory purposes.

Currently, the molecular weight (Mw) of a substance is used to determine whether it falls under the definition of a polymer or not. Mw is related to the number of monomers present in the polymer molecule, which must be ≥3. To determine the Mw, the OECD TG 118 is recommended to be applied, with gel permeation chromatography as the preferred method.^14^ In principle, this method uses the polymer molecule size (radius of gyration, *R*_g_) to determine their MW and Mw distribution, relying on the retention volumes of a set of relevant monodisperse polymer standards (typically polystyrene is used to obtain a universal calibration) under ideal conditions.^15^ To illustrate how the Mw relates to the polymer size, we used previously published information^16−20^ to plot the Mw of polymers versus their hydrodynamic diameter (2 × *R*_h_) (Figure 1). In some studies where R_g_ was reported for polymers,^21^ the *R*_h_ was calculated using the following formula:
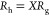
1where *X* is a proportionality constant which is assumed to be independent of Mw or solvent and was calculated from literature.^16^

For the polymers used in this example, the molecular size is below 100 nm, and hence, could be subject to regulation as nanomaterials. The molecular size of most plastic monomers in all dimension falls below the 1 nm (e.g., styrene: 6.8 × 4.4 Å^22^ and ethylene: 4.1 Å) lower limit for nanomaterials, and also fall below the threshold for polymers (<3).

## Recommendations

There is no credible scientific reason not to include nanoplastics in existing regulations as they meet all the criteria of chemicals and nanomaterials. Knowledge available on the toxicity of nanoplastics is a strong motivation for including intentionally produced nanoplastics in relevant regulations, as particle-specific hazards may be relevant. Future research should tease out hazard mechanisms of nanoforms of plastic separating physical (nanoparticle) from chemical (compositional) effects. Future regulatory updates for polymers should consider intentionally produced nanoplastics and whether data requirements should follow those for nanomaterials and/or polymers. Intentionally produced nanoplastics are a relatively minor emission source compared to incidental nanoplastics, and such emissions may be controlled by the application of safe-by-design principles in current regulatory frameworks or might be covered by microplastics regulations. Since MW and particle size are interconvertible it does not matter which parameter is chosen as the basis for regulation, but given the huge effort that has already gone into shaping REACH for nanomaterials, the practical approach would be to include nanoplastics in the nanomaterials regulation.
